# Histamine augments collagen content via H1 receptor stimulation in cultures of myofibroblasts taken from wound granulation tissue

**DOI:** 10.1007/s11010-020-03974-6

**Published:** 2020-11-23

**Authors:** Monika Wolak, Ewa Bojanowska, Teresa Staszewska, Lucyna Piera, Jacek Szymański, Jacek Drobnik

**Affiliations:** 1 Department of Behavioral Pathophysiology, Chair of General and Experimental Pathology, Medical University of Lodz, ul. Żeligowskiego 7/9, Lodz, Poland; 2 Laboratory of Connective Tissue Metabolism, Department of Pathophysiology, Chair of General and Experimental Pathology Medical University of Lodz, ul. Żeligowskiego 7/9, Lodz, Poland; 3 Central Scientific Laboratory, Medical University of Lodz, Lodz, Poland

**Keywords:** Collagen, Cell culture, Histamine, Myofibroblast, Fibrosis, Healing

## Abstract

The inflammatory reaction influences the deposition of collagen within wound granulation tissue. The aim of the present study is to determine whether histamine acting directly on myofibroblasts derived from wound granulation tissue may influence collagen deposition. It also identifies the histamine receptor involved in this process. The experiments were carried out on cells isolated from the granulation tissue of a wound model (a polypropylene net inserted subcutaneously to rats) or intact rat skin. Collagen content was measured following the addition of different concentrations of histamine and treatment with histamine receptor antagonists (ketotifen – H1 inhibitor, ranitidine – H2 inhibitor) and a histamine receptor H1 agonist (2-pyridylethylamine dihydrochloride).

The cells were identified as myofibroblasts: alpha-smooth muscle actin, vimentin, and desmin positive in all experimental conditions. Histamine increased the collagen level within both cell cultures, i.e., those isolated from granulation tissue or intact skin. It did not, however, influence the expression of either the collagen type I or III genes within the cultured myofibroblasts. Histamine activity was reduced by ketotifen (the H1 receptor inhibitor) and increased by the H1 receptor agonist, as demonstrated by changes in the levels of collagen in the myofibroblast culture. Histamine increased collagen content within the cultures, acting directly on myofibroblasts via H1 receptor stimulation.

## Introduction

Wound repair is a complex phenomenon comprising several interdependent processes such as hemostasis, inflammation, cell migration and proliferation, and protein synthesis, as well as wound contraction and remodeling. A fundamental element of the wound environment is constituted by the cells that participate in all repair processes, e.g., fibroblasts, myofibroblasts, epithelial cells, endothelial cells, and leukocytes. The cells are sources and targets of the signals derived from the wound milieu or the regulatory systems of the organism. The signal can be potentialized or inhibited in the wound environment; however, it then must be translated into intracellular information which can influence the metabolism of the cells. Through the activity of a number of regulatory processes, these phenomena act together to form a mature scar [1].

The inflammatory process in a wound is aimed at delivering leukocytes to the site of injury. These cells are responsible for the removal of bacteria and cellular debris, as well as the secretion of mediators involved in wound repair regulation. Thus, it is crucial that an appropriate inflammatory signal is produced to facilitate wound healing and scar development.

Mast cells play a key role in mediating inflammation by producing histamine. Higher numbers of mast cells have been found in patients with fibrotic diseases [2], and mast cells have been found to stimulate proliferation of co-cultured dermal fibroblasts following activation by IgE [3]. Sonicates of mast cells were also found to potentialize proliferation of fibroblasts derived from patients with Crohn’s disease [4]. However, histamine has also been shown to increase both fibroblast proliferation and collagen synthesis [5, 6]. Inhibition of histamine synthesis impaired wound healing of linear wounds, while elevated histamine release, facilitated by the application of compound 48/80, promoted healing [7].

Histamine exerts a vasodilatory effect and enhances the amount of blood going to the wound. It has also been shown to participate in the regulation of healing in vivo, with mast cell stabilization retarding the healing of open skin wounds [8]. The administration of histamine, or its liberators, accelerated the production and polymerization of collagen within granulation tissue in guinea pigs [9] and rats [10]. The healing rate has also been found to be inversely proportional to the level of histamine, which appears to regulate the progression of the healing process [11].

Thus, two hypotheses have been made to explain the effect of histamine on collagen synthesis. Firstly, it has been proposed that histamine encourages collagen synthesis by increasing the vasodilation of blood vessels within wounds and enhancing the blood supply to granulation tissue. Secondly, histamine may increase collagen synthesis by exerting a direct influence on the granulation tissue cells responsible for the synthesis of the extracellular matrix. Our earlier experiments performed on cells isolated from wound models confirmed that histamine accelerates the metabolism of the cells and increases TGFβ1 secretion [12]. This effect was not seen in cultures of naïve fibroblasts derived from the intact skin of rats [12]. Some other studies have shown that histamine may modulate collagen synthesis in human dermal fibroblast cultures [13] or in skin fibroblasts derived from guinea pigs [14]. Histamine applied at a concentration of 100 μM increased collagen type I content within subcutaneous human fibroblasts [15].

No studies have yet examined the accumulation of collagen by wound granulation tissue cultures under the influence of histamine. The aim of the present study is to determine the effect of different doses of histamine on the collagen content of wound-derived cells and to identify the receptor involved in the effect of histamine. This will be achieved by isolating cells from granulation tissue and identifying them by flow cytometry.

## Materials and method

### Animals and experimental design

Thirty male Wistar rats weighing 210 g ± 30 g were used for the wound healing experiments. The rats were maintained in plastic cages in controlled dark/light cycles (LD = 12 h:12 h) with water ad libitum and free access to food pellets (LSA, Bacutil, Poland). The Local Commission of Ethics in Lodz approved the protocol of the study. All animal experiments were conducted according to the humane guidelines specified in Polish animal protection legislation dated January 15, 2015.

### Wound model

In the left lumbar region of the anesthetized rat, a skin incision was made. Then, sterile polypropylene nets (3 × 2 cm) were implanted subcutaneously. The skin incision was closed by four silk sutures. After 4 weeks, the polypropylene meshes that had been grown over with granulation tissue were removed under aseptic conditions to set up the cell cultures. This model is recommended by Cohen [16] and was used in our earlier studies [8, 12, 17]. The implantation of the polypropylene mesh induces an immediate and local inflammatory response that decreases over time [18]; following this, fibroblasts appear and begin the synthesis of the extracellular matrix. The present model replicates the healing phenomenon.

### Cell isolation and culture

The granulation tissue obtained from the subcutaneously implanted meshes or intact skin of lumbar region of rats was stored in RPMI1640 medium (Gibco, Waltham, MA, USA) containing gentamicin (25 μg/ml, Gibco, Waltham, MA, USA) and amphotericin B (2.5 μg/ml, Gibco, Waltham, MA, USA). The material was homogenized and incubated in a 0.1% solution of collagenase (Sigma, St. Louis, USA) at 37 °C for 30 min. Then, the cells were washed. Culture of the adherent cells was performed in a CO_2_ incubator at 37 °C, under a 100% humidified atmosphere of 5% CO_2_ and 95% air. The experiments were carried out on cells after the second passage. The experimental culture was set up at an initial cell density of 8 × 10^3^/cm^2^. The cells were stained with trypan blue and counted in a Bürker chamber. The cells were cultured in DMEM (Gibco, Waltham, MA, USA) with 3% fetal bovine serum (Biowest, Nuaille, France), gentamicin (25 μg/ml), and amphotericin B (2.5 μg/ml). After a 6-day long culture, the cells were trypsynized, counted, and collected for flow cytometry or analysis of total collagen content.

A consecutive series of experiments which used cells derived from different rats was performed.

### Study design

The cells were treated with histamine (Sigma, St. Louis, USA): at concentrations of 5 × 10^−4^ M to 5 × 10^−5^ M for the granulation tissue-derived myofibroblasts, and 10^−4^ M, 10^−5^ M, and 10^−6^ M for cells from intact skin. The results obtained from the groups administered with histamine were compared with those from the untreated controls.

The second part of the experiment examined the effect of ketotifen (Sigma, St. Louis, USA). The cells were divided into four groups: a control group, a group treated with histamine (10^−4^ M) alone, a group treated with both histamine (10^−4^ M) and ketotifen (10^−3^ M), and another group treated with ketotifen (10^−3^ M). Ketotifen is mainly known as an H1 histamine receptor inhibitor. Although it has also been reported to inhibit histamine release from mast cells, this mechanism is irrelevant when ketotifen is administered to myofibroblast culture.

The third part of the experiments included four groups: untreated controls, those treated with histamine (10^−4^ M), those treated with both histamine (10^−4^ M) and ranitidine (10^−3^ M), and those treated with ranitidine alone (10^−3^ M). Ranitidine is an H2 histamine receptor inhibitor.

The H1 histamine receptor agonist 2-pyridylethylamine dihydrochloride (Sigma, St. Louis, USA) was applied to cultures at concentrations ranging from 10^−3^ M to 10^−7^ M. The results were compared with those from untreated controls.

### Flow cytometry

To verify phenotype of the isolated cells, the expression of α-smooth muscle actin, vimentin, and desmin was examined by using flow cytometry. Initially, the cells were washed three times with PBS. After fixation in Fixation Buffer (BD Biosciences, Franklin Lakes, NJ, USA) at 4 °C for 30 min, the cells were permeabilized in Perm Buffer (Biosciences, Franklin Lakes, NJ, USA) at 4 °C for 30 min. The cells were washed and stained using Stain Buffer (BD Biosciences, Franklin Lakes, NJ, USA): the probes were distributed into tubes containing 5 × 10^4^ cells/tube and staining was performed at 4 °C for 30 min. The following antibodies were applied for the staining: Vimentin LN-6, (Novus Biologicals, Littleton, CO, USA), Mouse IgM Isotype Control, (eBioscience San Diego, USA), Desmin DES/1711, (Novus Biologicals, Littleton, CO, USA), Mouse IgG1 Kappa Light Chain Isotype Control, (Novus Biologicals, Littleton, CO, USA), Alpha- Smooth Muscle Actin (Novus Biologicals, Littleton, CO, USA), and Mouse IgG2a Kappa Light Chain Isotype Control (Novus Biologicals, Littleton, CO, USA).

All applied antibodies were labeled with fluorescein isothiocyanate (FITC). A CytoFLEX cytometer (Beckman Coulter) was used for all measurements. The data were analyzed with Kaluza Analysis 2.1 Software (Beckman Coulter).

### Determination of collagen

The total collagen content of each sample was estimated by hydroxyproline evaluation. After drying, the cells were hydrolyzed by 6 M HCl for 24 h at 100 °C in a water bath. The hydrolysates were neutralized by 5 M NaOH, and 0.5 ml of each sample was diluted with redistilled water to 1 ml of final volume. Chloramine T was added to oxidize the hydroxyproline to pyrrole. The reaction was performed in a citrate buffer (pH = 6) for 20 min. Following this, 3.15 M perchloric acid was incubated with each sample for 5 min. Finally, the material was incubated with 20% p-dimethylaminobenzaldehyde (Sigma, St. Louis, USA) in a 60 °C water bath for 20 min. The optical density was measured at 560 nm on a spectrophotometer.

## Quantitative PCR

The experiment was composed of three steps: RNA isolation, transcription, and amplification. Total RNA Mini Kit (A&A Biotechnology, Gdynia, Poland) was used for isolation of the RNA from the cells. The process of transcription was performed with PrimeScript RT-PCR Kit (Takara, Kusatsu, Shiga, Japan). Gene expression was evaluated with Universal Probe Library (UPL) (Roche, Indianapolis, IN, USA) for the genes coding for collagen type I and III, and Hprt1 (hypoxanthine-guanine phosphoribosyltransferase), as well as *RPL13A* (60S ribosomal protein L13a), while RealTime ready Custom Single Assays (Roche, Indianapolis, IN, USA) were used for the *GAPDH* gene (glyceraldehyde 3-phosphate dehydrogenase). The genes for collagen type I and III were investigated, while *GAPDH*, *Hprt1*, and *RPL13A* were used as reference genes. For the collagen type I gene, GGGATTCCCTGGACCTAAAG was used as the F primer, GGAACACCTCGCTCTCCA as the R primer, and UPL probe #67; for collagen type III, TCCCCTGGAATCTGTGAATC was used as the F primer, TGAGTCGAATTG GG GAGAAT as the R primer and UPL probe #49. For Rpl13a, CCCTCCACCCTATGACAAGA was used as the F primer, GGTACTTCCACCCGACCTC as the R primer and UPL probe #74. Finally, for Hprt1, CTCCTCAGACCGCT TTTCC was used as the F primer, TCATAACCTGGTTCATCATCA as the R primer and UPL probe #95. Each sample was evaluated twice. The reaction was performed using Fast Start Essential Probe Master (Roche, Indianapolis, IN, USA) according to the following program: initial 10-min incubation at 95 °C, followed by 55 cycles of 10-s incubation at 95 °C, 30-s incubation at 60 °C, and 1-s incubation at 72 °C. Finally, a 30-s incubation at 40 °C was performed. LightCycler^®^ 96 software (Roche, Indianapolis, IN, USA) was used to calculate relative gene expression.

### Statistical analysis

The data were tested for normality using the Shapiro–Wilk test. As its distribution was found not to have a normal distribution, the data were tested using the non-parametric Kruskal–Wallis test. The differences between experimental groups were checked with Dunst’s test. Differences of *p* < 0.05 were assumed to be significant.

## Results

**Fig. 1:**
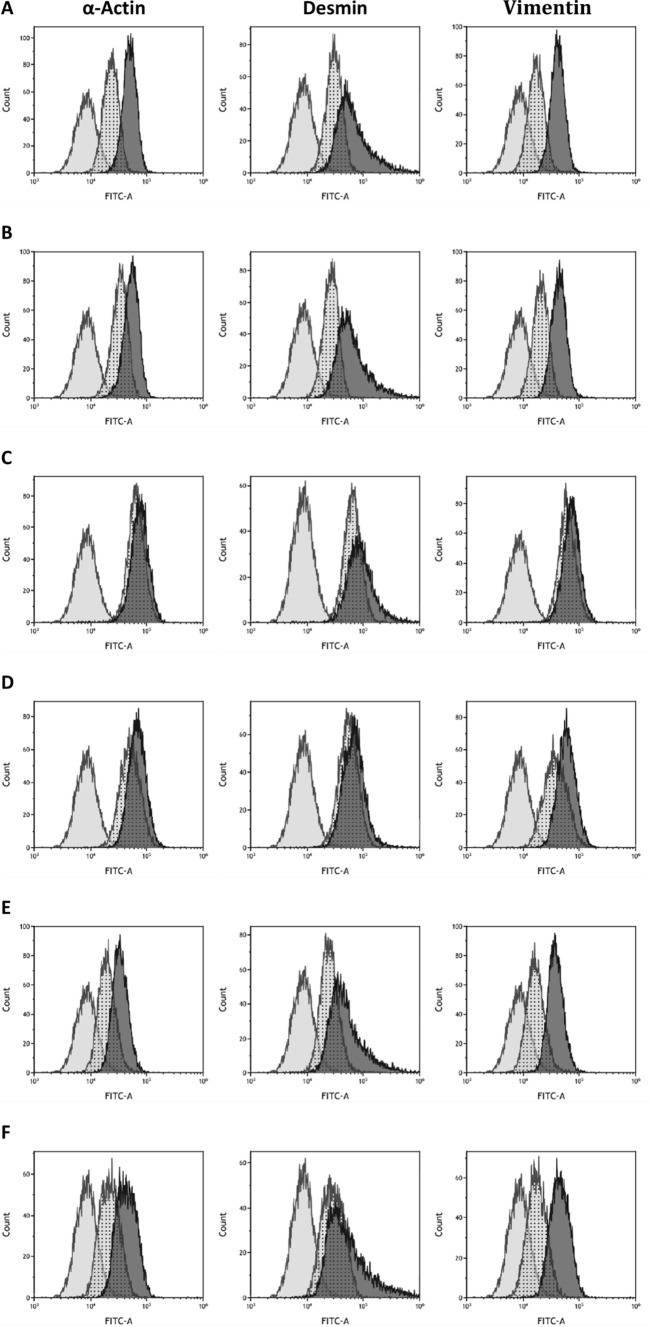
The density of α smooth muscle actin, desmin, and vimentin, (right curve) within cells derived from granulation tissue of wounds compared with an isotypic control (curve in the middle) and untreated cells of control (left curve) shown by flow cytometry profiles. The figures represent untreated cells (Fig. 1a), cells treated with histamine (Fig. 1b), ketotifen (Fig.1c), ranitidine (Fig. 1d), cultures treated with histamine and ketotifen (Fig.1e) as well as pyridylethylamine dihydrochloride (Fig. 1f). FITC-A represents the fluorescence intensity of fluorescein isothiocyanate (FITC) complexed with antibodies

The cells isolated from the granulation tissue of the wound or intact skin were α-smooth muscle actin, vimentin and desmin positive (Fig. 1). Moreover, the cells spread on Petri dishes had different shapes (longitudinal, fusiform, spindle, and stellate). These observations suggest that the isolated cells are myofibroblasts. Histamine, H1 agonist, and histamine with ketotifen did not appear to influence the phenotype of the isolated cells. Expression of α-smooth muscle actin, vimentin, and desmin is reduced by ketotifen and ranitidine (Fig. 1c and d).

**Fig. 2:**
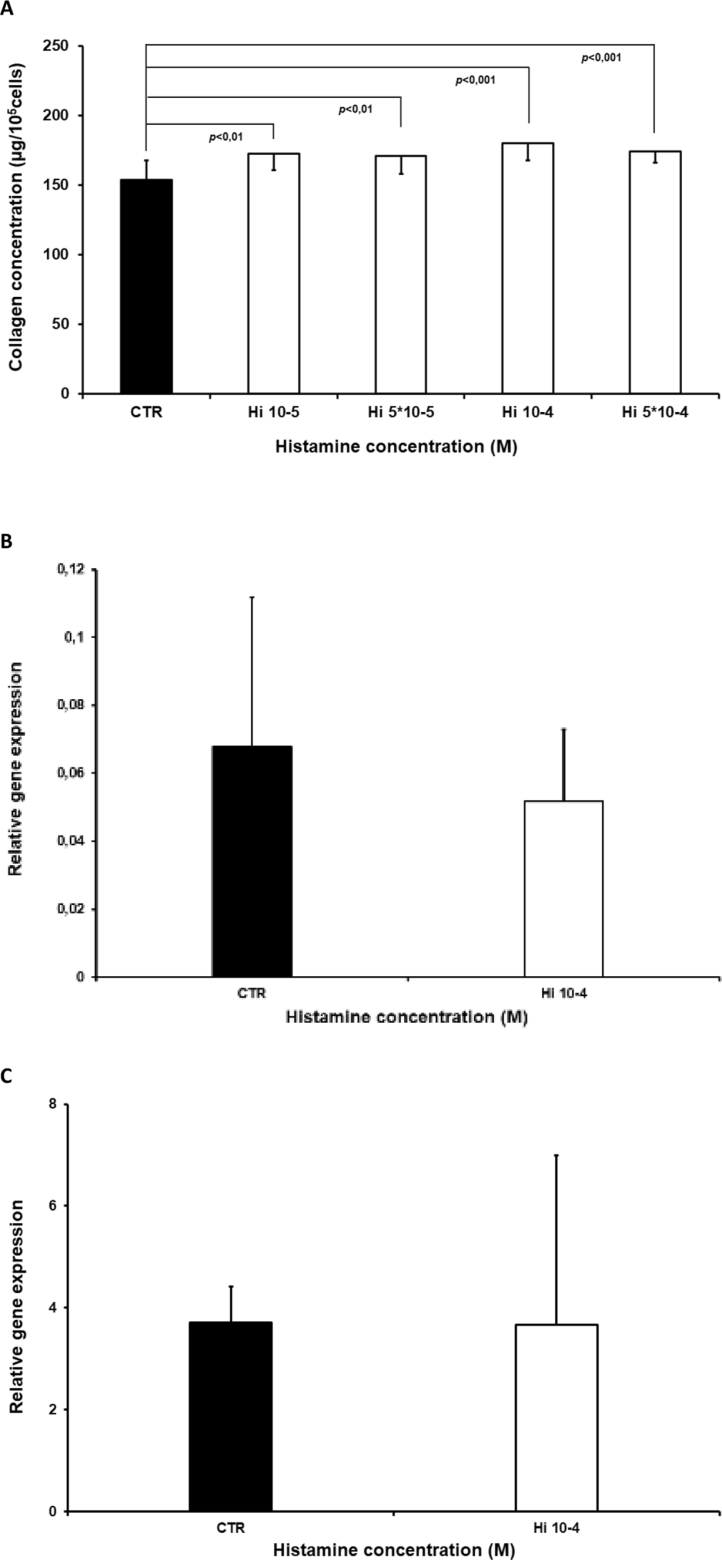
Collagen content (μg/10^5^ cells), (Fig. 2a) within myofibroblast cultures isolated from the granulation tissue of the wound, in the control group (CTR) and in cells treated with histamine at concentrations of 5 × 10^−5^ M (Hi5 × 10-5), 10^−5^ M (Hi10-5), 5 × 10^−4^ M (Hi5x10-4), and 10^−4^ M (Hi10-4). Each value expresses the mean of ten cultures + standard deviation (SD). Relative expression of α1 chain of procollagen type I (Fig. 2b) and type III (Fig. 2c) genes within myofibroblast cultures isolated from wound granulation tissue: control group (CTR) and cells treated with 10^−4^ M histamine (Hi10-4).

**Fig. 3:**
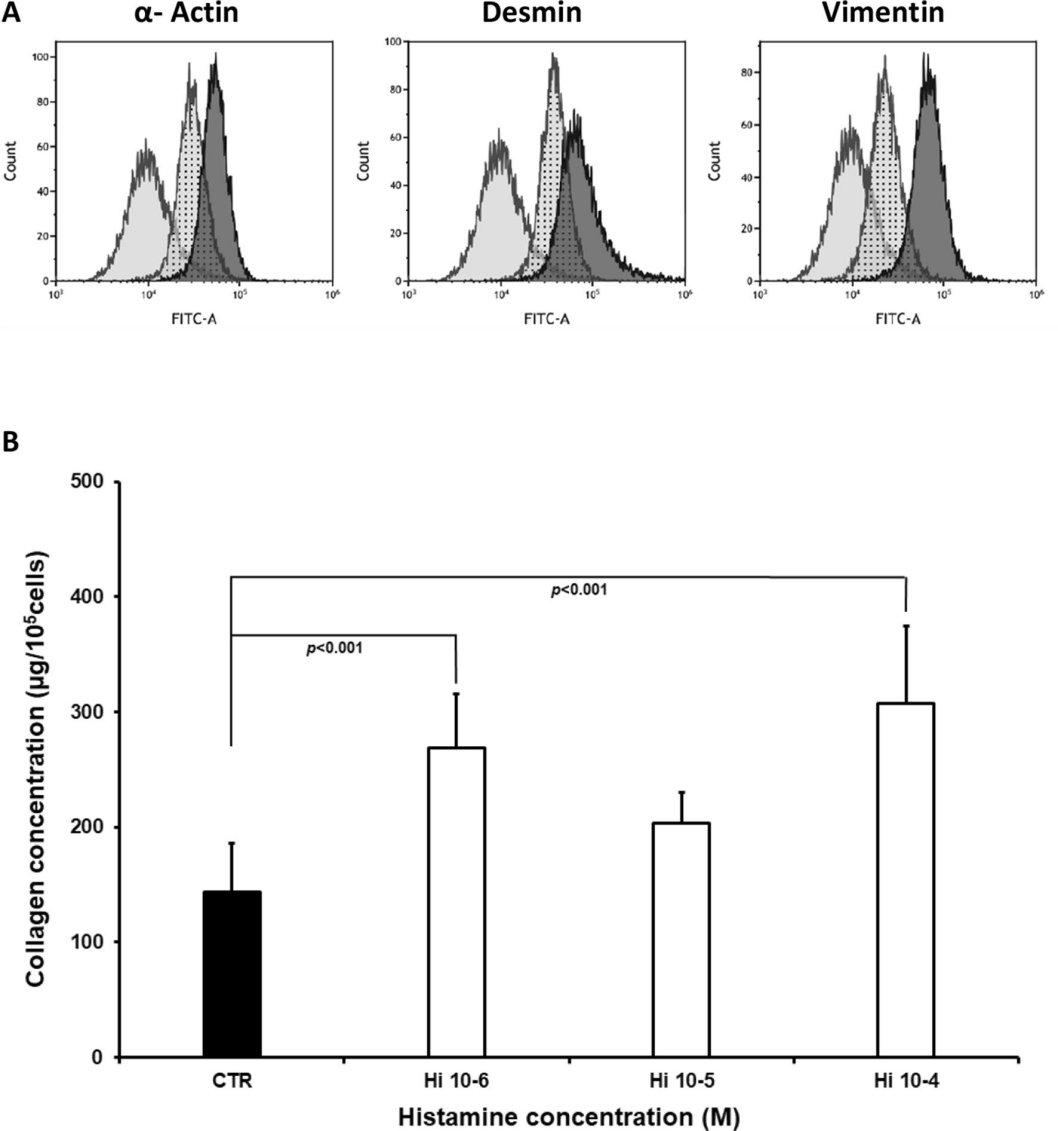
The density of α smooth muscle actin, desmin, and vimentin, (right curve) within cells derived from granulation tissue of wounds compared with an isotypic control (curve in the middle) and untreated control cells (left curve) shown by flow cytometry profiles. FITC-A represents the fluorescence intensity of fluorescein isothiocyanate (FITC) complexed with antibodies (Fig. 3a). Collagen content (μg/10^5^ cells) within myofibroblast cultures isolated from intact skin, in the control group (CTR) and in cells treated with histamine at concentrations of 10^−4^ M (Hi10-4), 10^−5^ M (Hi10-5), and 10^−6^ M (Hi10-6). Each value expresses the mean of ten cultures + standard deviation (SD), (Fig. 3b)

Myofibroblast cultures demonstrated increased collagen content compared to controls following treatment with histamine at concentrations of 5 × 10^−5^ M, 10^−5^ M (*p* < 0.01), 5 × 10^−4^ M and 10^−4^ M (*p* < 0.001) (Fig. 2a). Histamine did not influence the expression of either the collagen type I or III genes within the cultured myofibroblasts applied at 10^−4^ M (Fig.2b and c). In addition, histamine increased collagen content (Fig. 3b) in cultures of myofibroblasts (Fig. 3a) obtained from the intact skin. Significantly greater collagen content was reported in cultures treated with histamine at 10^−4^ M (*p* < 0.001) and 10^−6^ M (*p* < 0.04). An insignificant change was observed for 10^−5^ M.

**Fig. 4:**
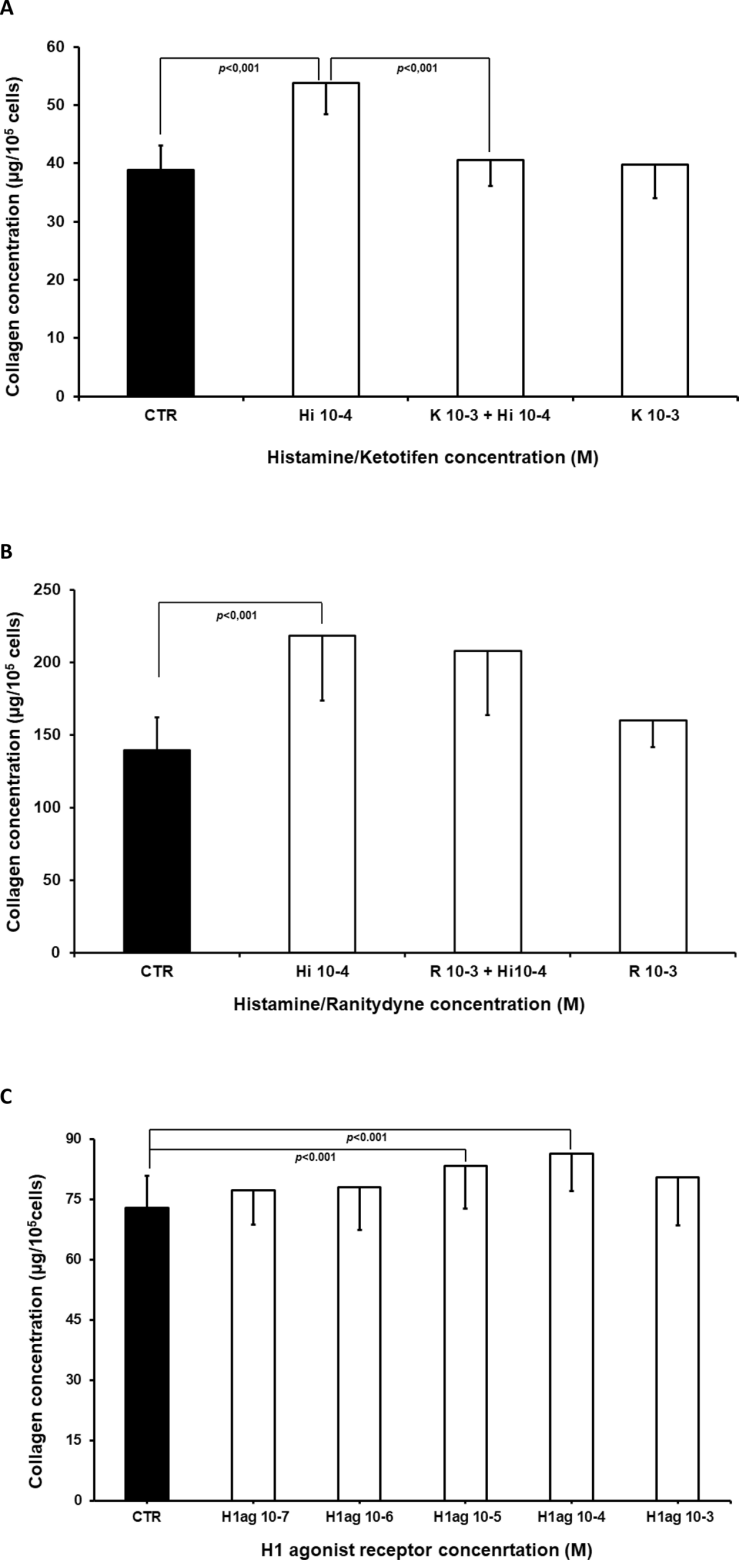
Collagen level (μg/10^5^ cells, Fig. 4a) in cultures of myofibroblasts from wound granulation tissue, of the control group (CTR) and in cells treated with histamine at a concentration of 10^−4^ M (Hi10-4), histamine (10^−4^ M) with ketotifen at a concentration of 10^−3^ M (K10-3+ Hi10-4), or ketotifen alone at a concentration of 10^−3^ M (K10-3), (Fig. 4a) Collagen content (μg/10^5^ cells Fig. 4b) in cultures of myofibroblasts from wound granulation tissue, of the control group (CTR) and in cells treated with histamine at a concentration of 10^−4^ M (Hi10-4), histamine (10^−4^ M) with ranitidine at a concentration of 10^−3^ M (R10-3+ Hi10-4), or ranitidine alone at a concentration of 10^−3^ M (R10-3). Collagen content (μg/10^5^ cells, Fig. 4c) in cultures isolated from wound granulation tissue, of the control group (CTR), and cells treated with pyridylethylamine dihydrochloride (H1 receptor agonist) at concentrations of 10^−7^ M (PD10-7), 10^−6^ M (PD 10-6),10^−5^ M (PD 10-5), 10^−4^ M (PD 10-4), and 10^−3^ M (PD 10-3). Each value expresses the mean of ten cultures + standard deviation (SD)

In the second part of the study (Fig. 4a), 10^−4^ M histamine treatment increased collagen content when compared with controls (*p* < 0.001). In addition, 10^−3^ M ketotifen, an H1 receptor inhibitor, significantly reduced the effect of histamine when compared to cells treated with histamine alone (*p* < 0.001); however, 10^−3^ M ketotifen treatment alone did not influence collagen content compared to controls. The increase in collagen content observed following treatment with 10^−4^ M histamine was not modified by 10^−3^ M ranitidine (Fig. 4b). No change in collagen content was observed following treatment with 10^−3^ M ranitidine alone (Fig. 4b).

Finally, the H1 receptor agonist, 2-pyridylethylamine dihydrochloride, applied at doses ranging from 10^−7^ M to 10^−3^ M, augmented collagen content within the cell cultures (*p* < 0.001) (Fig. 4c).

## Discussion

The cells isolated from the granulation tissue of the wound were identified as myofibroblasts based on their type of spread on Petri dishes and the intracellular expression of α-smooth muscle actin, vimentin, and desmin [19]. The mechanisms underlying the effects of both ketotifen and ranitidine on the expression of α-smooth muscle actin, vimentin, and desmin should be investigated in later study. Furthermore, the cells isolated from intact skin were also positive for α-smooth muscle actin, vimentin, and desmin; hence, it is possible that the skin fibroblasts were transformed into myofibroblasts when in contact with the stiff seeding substrate [20].

Histamine increased the collagen content of the myofibroblasts cultured from the wound granulation tissue when applied at doses ranging from 5 × 10^−5^ M to 5 × 10^−4^M. In addition, increased collagen content was also observed within the non-stimulated cells derived from intact skin following treatment with 10^−4^ M and 10^−6^ M histamine. Our results show that collagen content is elevated as a result of direct action of histamine, both in myofibroblasts and in cell cultures not stimulated by the wound.

Although this is the first such study to be performed on myofibroblasts derived from skin wounds, our findings are consistent with similar studies. For instance, histamine injected to rats or guinea pigs increased collagen content and increased its degree of polymerization within the wound [9, 10]. It has also been found to increase collagen type I synthesis within human fibroblast cultures when applied at concentrations between 10^−6^ M and 10^−4^ M [21]. Similar effects were observed in dermal fibroblast cultures. Both high (10^−6^ M – 10^−4^ M) and low (10^−9^ M) concentrations of histamine increased collagen synthesis [22]. Histamine application also increased procollagen type I carboxyterminal propeptide production [23]. Both histamine (10^−10^ M – 10^−5^ M) and mast cell sonicates were also found to increase the collagen content in rat myofibroblast cultures isolated from myocardial infarction scars [17]. Our previous findings indicate that histamine increased cellular metabolic activity in cells obtained from wound granulation tissue [12].

Our present findings indicate that histamine does not appear to have any influence on the transcription of collagen type I or III. However, it may influence collagen content by exerting a post-transcriptional influence on protein synthesis or breakdown.

The following experiments on histamine receptor inhibitors found that histamine increases collagen content in cell culture (Fig. 2). In addition, histamine-induced elevation of collagen content was blocked by ketotifen, an H1 histamine receptor inhibitor. The H2 inhibitor ranitidine was unable to modify histamine action on collagen content. Furthermore, the H1 receptor agonist, 2-pyridylethylamine dihydrochloride, augmented collagen levels in the myofibroblast cultures, mimicking the histamine effect (Fig. 4).

The above results clearly indicate that histamine increases collagen content in the myofibroblast cultures via stimulation of the H1 receptor and that the H2 histamine receptor is not involved in the regulation of collagen content in myofibroblast culture. H1 receptor expression has been previously reported in cells isolated from granulation tissue [12]. Some earlier in vivo studies also indicate that histamine receptors are involved in collagen content regulation within wounds [24]. Healing of incisional wounds of the skin has been found to be inhibited by two H1 receptor inhibitors: mepyramine and promethazine; however, no such activity was observed for the H2 receptor inhibitors [24]. The H1 receptor inhibitor, emedastine difumarate, also reduced histamine-induced elevation of collagen type I synthesis in dermal fibroblasts [13]. Both periostin and collagen gene expression in wild mice dermal fibroblast were found to be dependent on H1 receptor stimulation [25]. In addition, both the H1 receptor antagonist chlorpheniramine and H2 receptor antagonist cimetidine have been observed to reduce histamine-dependent collagen augmentation within fibroblast-like cell culture [14].

Collagen gel embedded in human lung fibroblasts was contracted by histamine applied at concentrations of 10^−6^ M and higher; the effect was inhibited by H1 receptor inhibitors [26]. In contrast, cimetidine, an H2 receptor inhibitor, was found to block the effects of histamine on collagen content in rat wound; however, mepyramine, an H1 receptor agonist, did not modify the collagen content [23]. In human fibroblasts, cimetidine blocked histamine-induced augmentation of collagen type I synthesis [22].

It has also been demonstrated that histamine increases secretion of TGFβ1 by cells isolated from wound granulation tissue obtained by a subcutaneously inserted polypropylene net and that secretion of this growth factor also appears to be regulated by H1 receptor stimulation. In addition, ketotifen was found to block histamine-induced augmentation of TGFβ1 concentration in the medium, while the H1 receptor agonist pyridylethylamine dihydrochloride increased the release of TGFβ1 [12]. These data support the hypothesis that histamine may act, at least partly, by inducing the release of TGFβ1. This growth factor may act in an autocrine or paracrine manner on myofibroblasts, thus increasing collagen accumulation in the cell cultures; however, more cytokines could be involved in the process. It is possible that histamine may increase the release of IL-6, IL-8, and CCL2 by orbital fibroblasts: their secretion was found to be inhibited by loratadine, an H1 receptor inhibitor, and the tested cells were found to express the H1 histamine receptor on the plasmalemma [27]. Histamine has also been found to increase VEGF synthesis in an H1 receptor-dependent manner [28]. It has also been reported that histamine increases the secretion of ATP from human subcutaneous fibroblasts via pannexin-1 hemichannels, causing mobilization of [Ca2+], and augmentation of collagen type I level within the culture. Histamine was also found to accelerate cell proliferation, measured by MTT, and these effects were dependent on the presence of H1 and P2 receptors [15]. Both these findings [15] and our earlier data [12] suggest that histamine exerts a complex influence on fibroblasts and myofibroblasts.

Histamine appears to exert a direct influence on myofibroblast cultures taken from wound granulation tissue, resulting in an elevation of collagen content within the cell culture, and this is realized by H1 histamine receptor stimulation. The presented mechanism could be a potential target for medicines recommended for therapy of non-healing wounds. Taken together, our findings indicate that the histamine receptor type involved in regulating collagen content is dependent on the target tissue and model of experiment.

## Data Availability

Data available in corresponding author.

## Code availability

(drob@op.pl).
